# Medical treatment of cystic echinococcosis: systematic review and meta-analysis

**DOI:** 10.1186/s12879-018-3201-y

**Published:** 2018-07-05

**Authors:** Virginia Velasco-Tirado, Montserrat Alonso-Sardón, Amparo Lopez-Bernus, Ángela Romero-Alegría, Francisco Javier Burguillo, Antonio Muro, Adela Carpio-Pérez, Juan Luis Muñoz Bellido, Javier Pardo-Lledias, Miguel Cordero, Moncef Belhassen-García

**Affiliations:** 1 Servicio de Dermatologia, Complejo Asistencial Universitario de Salamanca (CAUSA), Universidad de Salamanca, Paseo San Vicente 58-182, 37007 Salamanca, Spain; 2 Instituto de investigación Biomédica de Salamanca (IBSAL), Universidad de Salamanca, Paseo San Vicente 58-182, 37007 Salamanca, Spain; 3 Centro de Investigación de Enfermedades Tropicales de la Universidad de Salamanca (CIETUS), Universidad de Salamanca, Paseo San Vicente 58-182, 37007 Salamanca, Spain; 4 Área de Medicina Preventiva y Salud Pública, IBSAL, Universidad de Salamanca, C/Donantes de Sangre s/n. Campus Unamuno, 37007 Salamanca, Spain; 5 Servicio de Medicina Interna, CAUSA, Paseo San Vicente 58-182, 37007 Salamanca, Spain; 6 CIETUS, Paseo San Vicente 58-182, 37007 Salamanca, Spain; 7 Servicio de Medicina Interna, CAUSA, Universidad de Salamanca, Paseo San Vicente 58-182, 37007 Salamanca, Spain; 8 Departamento Química-Física, Facultad de Farmacia, Universidad de Salamanca, C/Donantes de Sangre s/n. Campus Unamuno, 37007 Salamanca, Spain; 9 Laboratorio de Inmunología Parasitaria y Molecular, CIETUS, IBSAL, Facultad de Farmacia, Universidad de Salamanca, C/Donantes de Sangre s/n. Campus Unamuno, 37007 Salamanca, Spain; 10 Servicio de Microbiología, CAUSA, Universidad de Salamanca, Paseo San Vicente 58-182, 37007 Salamanca, Spain; 11 Servicio de Medicina Interna, Hospital Marqués de Valdecilla, Avenida Valdecilla 25, 39008 Santander, Cantabria Spain; 12 Servicio de Medicina Interna, Sección de Enfermedades Infecciosas, CAUSA, Universidad de Salamanca, Paseo San Vicente 58-182, 37007 Salamanca, Spain

**Keywords:** *Echinococcus granulosus*, Cystic echinococcosis, Albendazole, Mebendazole, Praziquantel

## Abstract

**Background:**

Cystic echinococcosis (CE) is a well-known neglected parasitic disease. However, evidence supporting the four current treatment modalities is inadequate, and treatment options remain controversial. The aim of this work is to analyse the available data to answer clinical questions regarding medical treatment of CE.

**Methods:**

A thorough electronic search of the relevant literature without language restrictions was carried out using PubMed (Medline), Cochrane Central Register of Controlled Trials, BioMed, Database of Abstracts of Reviews of Effects, and Cochrane Plus databases up to February 1, 2017. All descriptive studies reporting an assessment of CE treatment and published in a peer-reviewed journal with available full-text were considered for a qualitative analysis. Randomized controlled trials were included in a quantitative meta-analysis. We used the standard methodological procedures established by the *Preferred Reporting Items for Systematic Reviews and Meta-Analyses* statement.

**Results:**

We included 33 studies related to the pharmacological treatment of CE in humans. Of these, 22 studies with levels of evidence 2 to 4 were qualitatively analysed, and 11 randomized controlled trials were quantitatively analysed by meta-analysis.

**Conclusions:**

Treatment outcomes are better when surgery or PAIR (*Puncture, Aspiration, Injection of protoscolicidal agent and Reaspiration*) is combined with benzimidazole drugs given pre- and/or post-operation. Albendazole chemotherapy was found to be the primary pharmacological treatment to consider in the medical management of CE. Nevertheless, combined treatment with albendazole plus praziquantel resulted in higher scolicidal and anti-cyst activity and was more likely to result in cure or improvement relative to albendazole alone.

**Electronic supplementary material:**

The online version of this article (10.1186/s12879-018-3201-y) contains supplementary material, which is available to authorized users.

## Background

Cystic echinococcosis (CE) is a neglected zoonosis caused by *Echinococcus* spp., mostly *Echinococcus granulosus*. Its huge socio-economic impact has been recently recognized. [1, 2]. CE has a worldwide geographical distribution, and the Mediterranean basin is considered an important endemic area [3]. Four treatment options are currently available: i) surgery, ii) PAIR, iii) chemotherapy with albendazole, mebendazole or other anthelmintic drugs, and iv) watch and wait for inactive or silent cysts. There is lack of evidence that supports treatment options [4]. This may be because several constraints in health-care system and CE is a chronic, complex disease [5]. Medical treatment is used for reducing cysts, decreasing infectivity and avoiding relapses. Besides, drugs are useful in disseminated or inoperable CE as the sole modality of treatment. To date, the medical treatment of CE is based on drugs of the benzimidazole family, usually albendazole [6, 7]. Over the last few years, praziquantel has been associated with albendazole [8, 9]. In addition, other drugs like nitazoxanide have also been used in disseminated CE [10]. Despite World Health Organization (WHO) recommendations, there is no standard for the medical management of CE, and variability exists in the timing of treatment initiation, dose and duration, which remain undefined.

The aim of this study is to analyse available data to answer the following clinical questions regarding the medical treatment of CE: i) Could pharmacological treatment improve the results of surgical interventions? ii) Is albendazole more effective than mebendazole? iii) Should albendazole be administered alone or in combination with praziquantel?

## Methods

### Search strategy and criteria for study selection

A systematic search of PubMed (Medline), Cochrane Central Register of Controlled Trials (CENTRAL), BioMed, DARE (Database of Abstracts of Reviews of Effects), and Cochrane Plus databases was conducted without language restrictions to identify studies that assessed the efficacy of medical treatment of CE and had been published up to February 1, 2017.

The following search key words and Boolean operators were entered: (*“cystic echinococosis”* OR *“hydatid disease”*, OR *“Echinococcus granulosus”*) AND (*“medical treatment”* OR *“albendazole”* OR *“mebendazole”* OR *“praziquantel”*) AND (*“randomized controlled trials”*) AND *“humans”* [AND NOT *“animals”*]. Additional records were also identified through other sources (UpToDate) (Additional file 1).

All relevant studies that reported the assessment of one modality of treatment or a comparison of two or several therapeutic methods to treat CE in humans and were published in a peer-reviewed journal with full text available were considered for analysis and classified according to levels of evidence and grades of recommendation proposed by the *Oxford Centre for Evidence-Based Medicine (OCEBM)* [11]. Data from editorials, letters to editors, reports of expert committees, and opinions of respected authorities based on clinical experience were excluded from the analysis because these designs do not have the same value, impact or power to make decisions or make recommendations. The results from non-randomized controlled trials, cohort or case-control analytic studies, prospective or retrospective case series, and literature reviews were qualitatively analysed but excluded from our quantitative meta-analysis. We included in the meta-analysis only studies from randomized controlled trials [Level of Evidence 1, Grade of recommendation A].

### Data extraction and quality assessment

After the relevant studies had been identified and selected, a systematic method was applied to data collection from each included study. The data collected were the first author’s name, year of publication, country of origin, study objective, study design, trial time period, number of patients, population characteristics, number of cysts, cyst location, mean cyst size, treatment, endpoint, main quantitative findings and conclusions. All relevant texts, tables and figures were reviewed for data extraction. A quality evaluation of each study was done, and conclusions were based on levels of evidence and grades of recommendation according to OCEBM [11].

The PRISMA *(Preferred Reporting Items for Systematic Reviews and Meta-Analyses)* statement [12] was used as a guide. Prespecified outcome-specific quality criteria were used to judge the admission of each qualitative and quantitative outcome into the appropriate analysis. Two investigators independently reviewed each eligible study and extracted the information and data necessary to carry out the qualitative analysis and the meta-analysis. Disagreements were resolved by consensus among all authors. The authors evaluated all randomized trials included in the meta-analysis to determine whether they were in accordance with the CONsolidated Standards Of Reporting Trials-CONSORT 2010 statement [12].

### Meta-analysis methods

Meta-analysis was performed utilizing the *Cochrane Review Manager (RevMan 5.3)* software. Statistical significance was defined at the level of 0.05. Recommendations of the PRISMA statement were considered as the outcome measure is dichotomic odds ratio was used as effect size. To combine studies to find a summary effect, we have resorted to the Mantel-Haenselz statistical weights. Heterogeneity across studies was followed with the *Cochrane Q-statistic* (whereby *p* ≤ 0.05 was considered statistically significant), and homogenicity of studies was rejected. The I^2^*-statistic* was also used to describe the percentage of total heterogenicity across studies. The following suggested cut-off points were used: I^2^ = 0–25%, no heterogeneity; I^2^ = 25–50%, moderate heterogeneity; I^2^ = 50–75%, large heterogeneity; I^2^ = 75–100%, extreme heterogeneity. A *fixed-effect model* was used if the *p*-value for Q was > 0.05 and I^2^ was < 50%. However, if both statistics rejected the homogeneity hypothesis, a *random-effect model* was used. The significance of the pooled odds ratio was evaluated with the Z test and its two-tailed p-value. Forest plots with odds ratios and their 95% confidence intervals were used to visualize all results. Unfortunately, the small amount of combinative data published to date did not allow any analysis of publication bias or validation, but the reported values in the present work can be considered consistent.

## Results

### Literature search

**Fig. 1:**
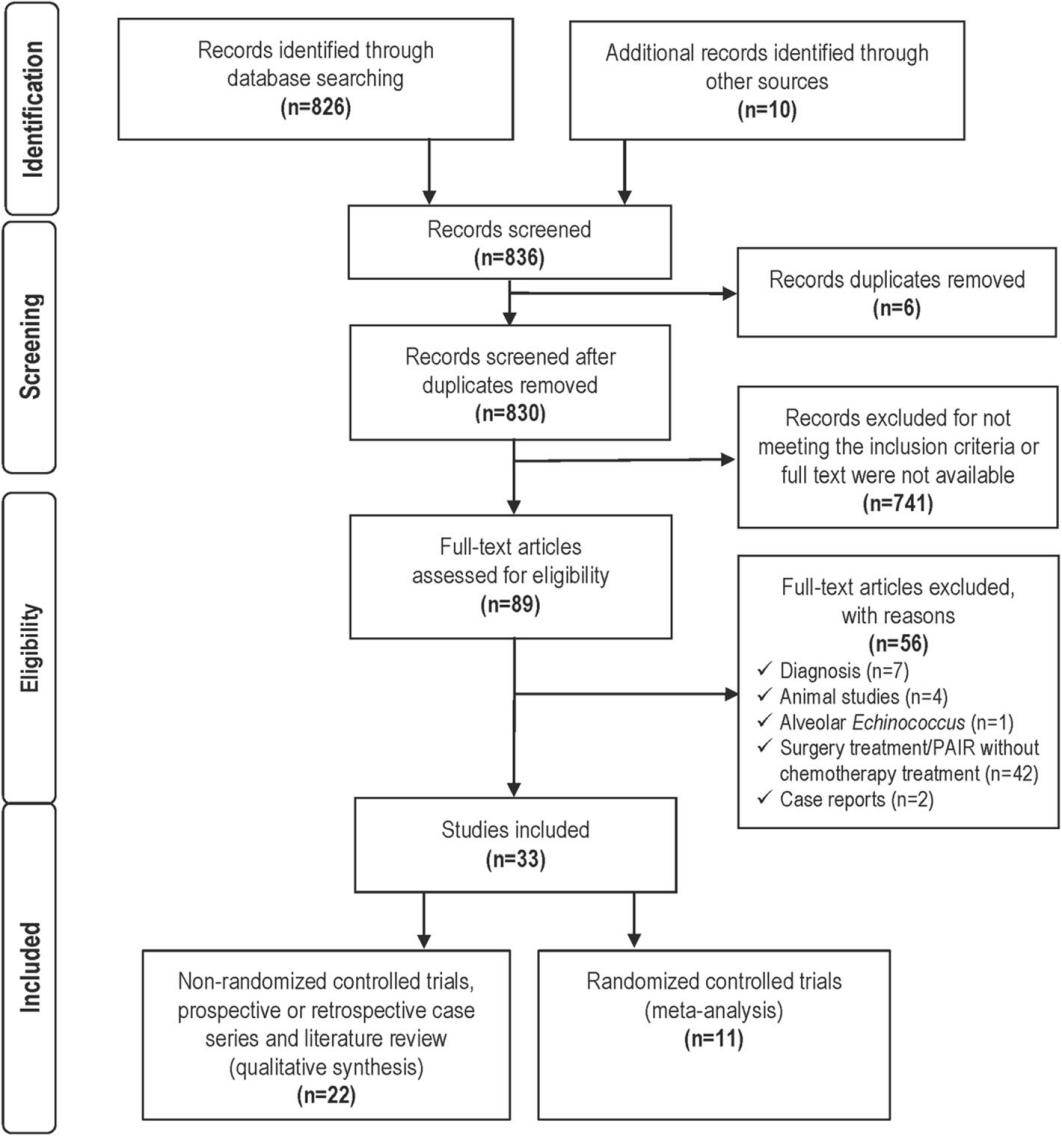
Flowchart of information through the different phases of the systematic review following the PRISMA recommendations

A PRISMA Flow diagram of the literature search is shown in Fig. 1.

By searching the electronic database, we identified 826 records related to the medical treatment of CE. Additionally, 10 records were identified through other sources. Six publications were removed because they were duplicate records. We screened 830 records, 741 of them were excluded for not meeting the inclusion criteria or full-text were not available. Eighty-nine full text articles were read in entirety. Fifty-six full-text articles were excluded for reasons including diagnosis (7), animal studies (4), studies of *Alveolar echinococcosis* (1), surgery treatment and PAIR without chemotherapy (42) and case reports/expert opinion [Level of Evidence 5] (2).

Thirty-three articles [6, 8–10, 13–41] related to medical treatment of CE in humans that met the inclusion criteria of CE treatment in humans were selected and classified by type of study design. Of them, 22 studies [6, 10, 13–32] non-randomized controlled trials, prospective or retrospective case series and literature reviews [Levels of Evidence 2 to 4] were qualitatively analysed, and 11 randomized controlled trials [8, 9, 33–41] [Level of Evidence 1] were quantitatively analysed by meta-analysis.

### Qualitative synthesis

This analysis included 22 studies [6, 10, 13–32] with levels of evidence below 1 (2 to 4): non-randomized controlled trial, one paper [13]; cohort study, one paper [21]; prospective descriptive study, six papers [15, 17, 22, 24, 25, 29]; retrospective study, six papers [16, 18, 23, 26, 28, 32]; and case series, two papers [10, 30] (Table 1 summarizes the main data of these studies in alphabetical order).

**Table 1:** Main characteristics of non-randomized controlled trials, prospective or retrospective case series included in the qualitative analysis

Author/s Year (Ref.no.)	Location, Country	Study design (follow-up period)	Participants	Sample size(N)	No. cysts	Cyst location	Objective	Anthelminthic drugs	Results/Conclusions
Aktan AO, et al. 1996 [13]	Istanbul, Turkey	A non-randomized controlled trial	Adult patients	70	89	Liver	To evaluate the effect of preoperative ABZ* treatment (3-weeks) in two groups: 1st group *(experimental group)* ABZ 3 weeks before surgery, 2nd group *(control group)* surgery (no preoperative treatment).	ABZ	The ICP values of viable cysts in the 1st group were significantly lower (*p* < 0.05). The number of non-viable cysts was also significantly higher in the 1st group (*p* < 0.05). ABZ has proved to be effective in decreasing the viability of liver hydatid cysts when given for 3 weeks preoperatively.
Di Matteo G*,* et al. 1996 [15]	Rome, Italy	A prospective, descriptive, non-comparative study (1985–1992)	Adult patients (mean age, 42)	95	No data	Liver	To show that radical surgery is most effective when it is associated with medical therapy of benzoimidazole drugs (MBZ) pre- and post-operatively.	MBZ*	The most effective treatment for echinococcus cystic disease of the liver is radical surgery. Results are best when surgery is combined with medical therapy of benzoimidazole drugs (MBZ) given pre- and post-operatively.
Doğru D, et al. 2005 [16]	Ankara, Turkey	A retrospective study	Pediatric patients	82	102	Lung	To demonstrate the safety and efficacy of medical treatment.	MBZ vs ABZ	The cure and the failure rates were statistically insignificant in cysts treated with MBZ and ABZ; however statistically significantly more cysts were improved with ABZ. The results were statistically insignificant between continuous and cyclic ABZ treatment. There was a positive, weak and statistically significant correlation between the cyst size and treatment results. These results cannot recommend a standard treatment regimen as the duration of treatment should be individualized for each patient.
el-Mufti M, et al. 1993 [17]	Benghazi, Libya	A prospective, descriptive, non-comparative study	Adult patients	40	63	Multi-organ	To assess the effectiveness of ABZ before surgery.	ABZ	It is suggested that patients suffering from uncomplicated hydatid disease should be given the benefit of a trial course of ABZ therapy before surgery.
Ghoshal AG, et al. 2012 [18]	Kolkata, India	A retrospective study (5 years)	Adult patients	106	No data	Lung	To determine the presentation, treatment (ABZ and surgery) and outcome of hydatid disease of lung.	ABZ	Surgery is a safe and effective way of treatment for thoracic hydatid cyst along with perioperative ABZ therapy. There is a scope for chemotherapy with ABZ in inoperable cases.
Larrieu E, et al. 2004 [21]	Rio Negro, Argentina	A prospective cohort study (5–6 years)	Pediatric patients	5745 *Exposed cohort* = 4644 *Unexposed cohort* = 1101	No data	Abdominal	To evaluate the results of a program carried out in endemic areas of the Province of Río Negro, Argentina, during the years 1997–2002 in asymptomatic children, screnning.	ABZ	Treatment with ABZ confirmed its action in modifying the prognosis of CE, presenting positive effects in 76% of patients receiving the drug. None of the treated cases required surgery. The combination of ultrasonographic screening and ABZ treatment showed promising results.
Li T, et al. 2011 [22]	Sichuan, China	A prospective, descriptive, non-comparative study	Adult patients	49	No data	Abdominal	A post-treatment follow-up study was carried out to assess the effectiveness of community based use of cyclic ABZ treatment in Tibetan CE cases.	ABZ	Cyclic ABZ treatment proved to be effective in the great majority of CE, but periodic abdominal ultrasound examination was necessary to guide appropriate treatment. Serology with recombinant antigen B could provide additional limited information about the effectiveness of ABZ in CE cases. Oral ABZ for over 18 months was more likely to result in CE cure.
Mikić D, et al. 1998 [23]	Republic of Serbia	A retrospective study	Adult and pediatric patients (female age range, 9–83; males age range, 6–72)	119	No data	Liver	To value the efficacy of ABZ and surgery.	ABZ	Surgical removal of the cyst takes a leading place in the treatment of hepatic echinococcosis. However, in well-selected cases and in the patients with high surgical risk, anthelminthic therapy and PD of echinococcus cyst are of more significance.
Nahmias J, et al. 1994 [24]	Moztkin, Israel	A prospective, descriptive, non-comparative study (3–7 years)	Adult patients	68	No data	Multi-organ	To assess long-term efficacy of ABZ.	ABZ	Follow-up for 3–7 years showed that this treatment alone eradicated the cysts in many patients; in most of the remainder, disease progression stopped. No patient worsened but a recurrence occurred in two patients at about 56 months.
Perez Molina JA, et al. 2011 [10]	Madrid, Spain	A case series	Adult patients (age range, 27–68)	7	No data	Multi-organ	To describe the clinical effectiveness and tolerability of nitazoxanide, combined with ABZ, with or without PZQ*, in patients affected by disseminated chronic CE.	ABZ vs ABZ + PZQ	Nitazoxanide combination therapy seems to be active for disseminated CE affecting soft tissues, muscles, or viscera, and apparently it has no role in chronic and extensive bony lesions.
Redzić B, et al. 1995 [24]	Republic of Serbia	A prospective, descriptive non-comparative study (from 1989 to 1993)	Adult patients	73	No data	Liver	To value the efficacy of PZQ.	PZQ	The drug treatment was the therapy of choice in patients with *Echinococcus granulosus*. It should be given prophylactically, preoperatively, to sterilize the cyst and also as a curative treatment.
Salinas JL, et al. 2011 [26]	Lima, Perú	A retrospective study (from January 1997 to December 2007)	Adult patients (mean age at diagnosis, 51 ± 14)	27	No data	Liver	To ascertain factors associated with the success of ABZ in the treatment of non-complicated hepatic CE, and to establish the frequency of long-term worsening and recurrence of disease after treatment completion in Peru.	ABZ	Long-term hepatic CE treatment outcomes and the success rate of ABZ were modest (3 cycles are few and needed treatment 6–12 months). It’s necessary to investigate into alternate therapeutic strategies for this neglected disease.
Tarnovetchi C, et al. 2010 [28]	Romania	A retrospective study (2004–2009 and 2000–2009)	Pediatric patients (age range, 2–17)	111	No data	Abdominal	To value the efficacy of ABZ and surgery (Lagrot partial pericystectomy).	ABZ	The treatment includes both surgical and medical means. There is a relatively high rate of postoperative complications (although some of them being minor) in 31 patients.
Todorov T, et al. 1992 [29]	Sofia, Bulgaria	A prospective descriptive study	Adult and pediatric patients (age range, 6–70)	51 (28 MBZ, 23 ABZ)	No data	Multi-organ	To test the efficacy of MBZ and ABZ.	MBZ or ABZ	Treatment with MBZ was successful in 8 (28.6%), partially successful in 8 (28.6%) and unsuccessful in 12 (42.8%). Treatment with ABZ was successful in 10 (43.5%), partially successful in 10 (43.5%) and unsuccessful in 3 (13.0%).
Yasawy MI*,* et al. 1993 [30]	Riyah, Saudi Arabia	A case series	Adult patients	4	No data	Pelvic, abdominal and thoracic	To value the response to combined medical treatment (ABZ and PZQ).	ABZ plus PZQ vs ABZ	This preliminary report shows that the response to combined treatment is better and much quicker compared to ABZ alone.
Yilmaz Y, et al. 2006 [32]	Van, Turkey	A retrospective study (10 years)	Adult and pediatric patients	372 (of them, 8 urinary hydatid disease)	No data	Liver, spleen, brain and kidneys(7)-retrovesical area(1)	To discuss therapeutic options and treatment results according to current literature.	ABZ	Treated surgically (271 cases) and drained percutaneously (99 cases). Kidneys were removed totally (4 cases), cystectomy and omentoplasty was performed in one case. ABZ was administered to 192 patients.

^*^ABZ Albendazole, *PZQ* Praziquantel, *MBZ* Mebendazol

We also included four papers corresponding to literature reviews [14, 20, 27, 31] and two systematic reviews [6, 19] in this qualitative analysis (Table 2).

**Table 2:** Main characteristics of the reviews included in the qualitative analysis

Author/s Year (Ref.no.)	Location, Country	Study design	Participants	Sample size (N)	No. cysts	Cyst location	Objective	Anthelminthic drugs	Results/Conclusions
Bygott JM, et al. 2009 l [14]	London, England	A literature review	In vitro/vivo animal studies, human studies	No data	No data	Liver, lung, intra-abdominal	To review the evidence on the use of PZQ in treatment of cystic hydatid disease from in vitro and in vivo animal studies, human clinical studies and human case reports.	PZQ	Insufficient published evidence to support a clear recommendation for the use of PZQ in prolonged chemotherapy for established hydatid disease for which surgery is not indicated or in severe disseminated disease and further work is necessary.
Horton RJ. 1997 [19]	Brentford, UK	A systematic review	Adult and pediatric patients (age range, 6–83)	3760	No data	Principally in the liver, with lung infection being the second most common	To review the efficacy and safety of ABZ obtained in the last 12 years.	ABZ	ABZ has been shown to be a useful advance in the management of CE both when used as sole treatment or as an adjunct to surgery or other treatments. Efficacy seems to increase with exposure up to 3 months in the commoner cyst sites.
Kern P, et al. 2003 [20]	Ulm, Germany	A literature review	Adult and pediatric patients	No data	No data	Liver, lung, kidney, spleen, muscles, abdominal and pelvic cavity,…	To review clinical presentation and medical treatment vs conservative treatment and outcome *Echinococcus granulosus* infection.	ABZ or MBZ or PZQ	Of major importance in the management of CE is long-term observation and longitudinal monitoring. Liver cysts relapse more frequently than do cysts at other sites, presumably because of greater proliferative potential of the metacestode tissue remaining in the hepatic environment. Further cycles of benzimidazole treatment of patients with recurrences were again well tolerated and effective. It was suggested that the higher metabolic activity of relapsed cysts makes them more susceptible to the action of benzimidazole carbamates.
Stamatakos M, et al. 2009 [27]	Athens, Greece	A literature review	Adult and pediatric patients	No data	No data	Liver, lung, and peritoneal cysts	To clarify anthelminthic treatment as an alternative hydatic cyst therapy, its indications and contraindications.	ABZ or MBZ	ABZ and MBZ have a favourable effect in patients suffering from multiorgan and multicystic disease, in inoperable primary liver or lung echinococcosis, and they can also prevent secondary echinococcosis. Chemotherapy is contraindicated for large cysts that are at risk to rupture and for inactive or calcified cysts. The main adverse events are related to changes in liver enzyme levels. The best efficacy is observed with liver, lung, and peritoneal cysts. Certain various factors influence the therapeutic results of medical treatment. The vast majority of the recurring cysts show good susceptibility to re-treatment.
Stojkovic M, et al. 2009 [6]	Heidelberg, Germany (6 Centers: Rome, Bulgaria, Romania, Palermo, Greece, Turkey)	A systematic review	Adult and pediatric patients	711	1159	Liver and peritoneal cysts	To describe cyst outcome after initiation of benzimidazole treatment, with outcome defined by cyst stage determined by ultrasound following the WHO classification of 2001.	ABZ or MBZ	The overall efficacy of benzimidazoles has been overstated in the past. There is an urgent need for a pragmatic randomised controlled trial. The clarification of the efficacy of benzimidazoles in CE treatment is of paramount importance since benzimidazoles are the only drugs currently available to treat this neglected disease.
Yasawy MI 2001 [31]	Saudi Arabia	A literature review	Clinical cases and animal studies	No data	No data	Multi-organ	To review the efficiency of benzimidazole (ABZ) and isoquineline (PZQ).	ABZ or PZQ	Combination therapy is more effective and requires a shorter period of treatment than ABZ alone. Pre- and postoperative prophylactic therapy reduce risk of spillage and dissemination during surgery and percutaneous aspiration.

^*^ABZ Albendazole, *PZQ* Praziquantel, *MBZ* Mebendazol

Chronologically, the oldest publication dates back to 1992 from Todorov et al. [29], while the most current was published in 2012 by Ghoshal et al. [18].

The geographic location of studies is varied: England (UK) [14, 19], Germany [6, 20], Italy [15], Spain [10], Greece [27], Yugoslavia [23, 25], Romania [28], Bulgaria [29], Turkey [13, 16, 32], Israel [24], Saudi Arabia [30, 31], Libya [17], India [18], China [22], Argentina [21] and Peru [26].

In relation to the study population, ten papers correspond to studies in adult patients [10, 13, 15, 17, 18, 22, 24–26, 30], three papers are studies in pediatric patients [16, 21, 28], seven studies are in both adults and pediatric patients [6, 19, 20, 23, 27, 29, 32], and two literature reviews covered both animal and human studies [14, 31]. The sample sizes of the studies were very different; 5745 school-age children participated in a study cohort [21], 3760 adult and pediatric patients were included in a systematic review [19], and 7 [10] and 4 [30] adult patients were included in the case series. Other sample sizes were 711, 372, 119, 111, 106, 95, 82, 73, 70, 68, 51, 49, 40 and 27 patients.

Relative to cyst location, the studies principally analysed multi-organ/abdominal cysts [6, 10, 14, 17, 19–22, 24, 27–32], liver [13, 15, 23, 25, 26] and lungs [16, 18].

The drug used in most antihelmintic therapies is albendazole [6, 10, 13, 16–24, 26–32], alone or combined with mebendazole [6, 15, 16, 20, 27, 29] or praziquantel [10, 14, 20, 25, 30, 32]. All studies are coincident in terms of their results.

From a qualitative assessment of the 22 studies, treatment outcomes are better when surgery or PAIR is combined with pharmacological therapy of benzimidazole drugs given pre- and/or post-operation. Based on a qualitative synthesis, there was a positive and statistically significant association between cyst size and treatment results. More cysts showed statistical improvement with albendazole than with praziquantel or mebendazol. Albendazole combined with mebendazole or praziquantel performed better than albendazole alone.

### Quantitative synthesis using meta-analysis

The meta-analysis included 11 randomized controlled trials [8, 9, 33–41] [Level of Evidence 1, Grade of recommendation A]. The main methodological characteristics of these studies are presented in Table 3, and therapeutic findings are shown in Table 4**.** Chronologically, the oldest publication dates back to 1986 (Davis et al. [34]), while the most current was published in 2011 (Shams-UI-Bari et al. [41]). The geographic location varies, including Turkey [33], Spain [9, 37], Switzerland [34, 35], Italy [36], Iran [38, 39], India [40, 41] and Saudi Arabia [8].

**Table 3:** Main methodological characteristics of randomized controlled trials included in the quantitative analysis *(meta-analysis)*

Author/s. Year (Ref.no.)	Location Country	Objective^a^	Study design	Trial time period	Participants	Sample size (N)^b^	No. Cysts	Patients characteristics^c^				
Bildik N, et al. 2007 [33]	Kartal-Istanbul, Turkey	To evaluate the efficacy of preoperative ABZ therapy	A randomized controlled trial	1998–2003	Patients with isolated hydatid cysts of the liver	84	84	Sex (M/F), 36/48 Range age (*yr*), (14–67)				
								Group I	Group II	Group III	Group IV-control group	
								*n* = 21 No.cyst = 21	*n* = 21 No.cyst = 21	*n* = 21 No.cyst = 21	*n* = 21 No.cyst = 21	
Cobo F, et al. 1998 [9]	Pamplona-Navarra, Spain	To compare the effects of a combined medication of ABZ plus PZQ vs ABZ alone in the preoperative treatment	A randomized controlled trial	1990–1997	Patients with intra-abdominal hydatidosis	62 → 47	No data			Group I	Group II	Group III
								n Sex (M/F) Age (*yr*, mean ± SD[range]) Cyst/patient (mean[range])		19 → 12 6/6 47.9 (19–67) 1.17(1–3)	17 → 14 11/3 51.1 (31–70) 1.57 (1–4)	26 → 21 9/12 46.9 (18–75) 1.43 (1–5)
Davis A, et al. 1986 [34]	WHO, Geneva, Switzerland	*First phase:* Studies coordinated by the WHO were conducted in seven clinical centers on the chemotherapy of human echinococcosis with MBZ, ABZ and FBZ.	A multicenter randomized clinical trials (5 clinical centers, Beirut, Paris, Rome, Sofia and Zurich)	1982–1984	Adults patients, mainly, only 7% below 15 years	121	121			MBZ	FBZ	ABZ
								*n* = 121 Sex (M/F) = 63/58 No.cyst = 402		85 38/47 348	6 3/3 18	30 22/8 36
Davis A, et al. 1989 [35]	WHO, Geneva, Switzerland	*Second phase:* To value the efficacy of ABZ and MBZ in human CE coordinated by WHO.	A multicenter randomized clinical trials (4 clinical centers, Beirut, Paris, Rome and Sofia)	1985–1987	Adults patients, mainly, only 4% below 15 years	176 → 112	106			ABZ	MBZ	
								*n* = 112 Sex (M/F) = 47/65 Follow-up < 12 months = 44 Follow-up > 12 months = 68 No.cyst patients > 12 months = 106		67 27/40 21 46 76	45 20/25 23 22 30	
Franchi C, et al. 1999 [36]	Rome, Italy	To evaluate the results obtained during long-term follow-up of a series of patients treated with benzimidazole carbamate	A randomized controlled trial	1982–1997	Patients with hydatidosis located in various body organs	448	929	Sex (M/F), 191/257 Age (*yr*, mean[range]), 52 (4–86) Follow-up (months, mean[range]), 22 (12–170)				
									MBZ		ABZ	
								n No.cyst	125 289		323 640	
Gil-Grande LA, et al. 1993 [37]	Madrid, Spain	To assess the efficacy and safety of ABZ in a medical treatment	A randomized controlled trial	1987–1991	Patients with intra-abdominal hydatid disease	66 → 55	55		Group A		Group B	Control gr.
								n Sex (M/F) Age (*yr*, mean ± SD) No.cyst	18 10/8 41.7 ± 14.2 18		19 12/7 47.3 ± 13.9 19	18 9/9 41.2 ± 17.3 18
Keshmiri M, et al. 1999 [38]	Mashhad, Iran	To compare the effects of ABZ vs placebo in the treatment of hydatid cysts	A triple-blind parallel randomized clinical trial	1994–1995	Patients with hydatid cysts of the lung/pulmonary echinococcosis	20^All p.^ 15^Treat.^	179^All p.^ 150^Treat.^		Treatment group		Placebo group	
									All p.^d^	Treat.^d^	All p.^d^	Treat.^d^
								n Sex (M/F) Age (*yr*, mean ± SD) No.cyst Cyst/patient	14 8/6 41 ± 15 137 12.2 ± 13.4	11 5/6 40 ± 17 124 16.3 ± 13.9	6 3/3 39 ± 17 42 10.8 ± 13.7	4 3/1 45 ± 17 26 8.8 ± 7.6
Keshmiri M, et al. 2001 [39]	Mashhad, Iran	To evaluate the effect of ABZ on hydatid disease	A double-blind parallel-group randomized clinical trial	1994–1995	Patients with hydatid cysts of the lung and abdomen (including liver)	29^All p.^ 21^Treat.^	240 ^All p.^ 203^Treat.^		Treatment group		Placebo group	
									All p.^d^	Treat.^d^	All p.^d^	Treat.^d^
								n Sex (M/F) Age (*yr*, mean ± SD) No.cyst Cyst/patient	22 11/11 41.4 ± 15.9 191 8.6 ± 9.0	17 7/10 40.5 ± 17.3 172 9.8 ± 9.9	7 4/3 35.4 ± 18.3 49 7.1 ± 6.5	4 3/1 45.5 ± 17.4 31 7.8 ± 6.1
Khuroo MS, et al. 1993 [40]	Srinagar, Kashmir, India	To compare the safety and efficacy of percutaneous drainage (PD) with ABZ therapy	A randomized controlled trial	1989–1992	Patients with hepatic hydatid cysts	30	33		PD		ABZ-PD	ABZ
								n Sex (M/F) Age (*yr*, mean ± SD) (Range age) No.cyst Size Ø (*cm*, mean ± SD) Volume (*cm*^*3*^, mean ± SD)	10 4/6 36.7 ± 12.3 (12–55) 10 9.2 ± 4.4 686 ± 651		10 3/7 41.3 ± 14.9 (12–64) 12 10.8 ± 3.0 835 ± 528	10 4/6 39.5 ± 14.4 (18–60) 11 8.8 ± 4.5 642 ± 717
Mohamed AE, et al. 1998 [8]	Riyadh, Saudi Arabia	To evaluate the effect of different regimens of medical treatment	Two prospective randomized intervention studies	1st study, 1985–1990 2nd study, 1990–1998	Adult Saudi patients with hydatid disease at the Armed Forces Hospital	1st, 22 2nd, 19, Total,41	No data	1st, ALB		2nd, ABZ+PZQ		
								*n* = 22		*n* = 19		
Shams-UI-Bari, et al. 2011 [41]	Srinagar, Kashmir, India	To assess the effect of preoperative ABZ therapy on the viability of protoscoleces at the time of surgery	A randomized controlled trial	2002–2003 + follow-up 5 years	Patients with diagnosis of hydatid liver disease	72	72	Sex (M/F), 39/33 Range age (*yr*), (17–66)				
									Group A-Surgery		Group B-ABZ+surg+ABZ	
								n Sex (M/F) Age (*yr*, mean ± SD[range]) No.cyst	36 19/17 36.75 ± 11.34(16–64) 36		36 20/16 36.78 ± 11.79(17–62) 36	

^a^*ABZ* Albendazole, *PZQ* Praziquantel, *MBZ* Mebendazol, *FBZ* Flubendazole

^b^N start of the study→N the end of the study

^c^M/F, Male/Female ratio

^d^All patients, Completed treatment

**Table 4:** Main therapeutic findings and conclusions of randomized controlled trials included in the quantitative analysis *(meta-analysis)*

Author/s. Year(Ref.no.)	Cyst location	Mean cyst size(cm)	Treatment^a^	Endpoint	Main quantitative findings*		
Bildik N, et al.*......* 2007 [33]	Liver	Non-registered information	G-I: ABZ (10 mg/kg/day 1 month before surgery) G-II: ABZ (10 mg/kg/day 2 months before surgery) G-III: ABZ (10 mg/kg/day 3 months before surgery) G-IV (control gr.): surgery (no preoperative therapy)	Viability of the scoleces		Intact	Dead
					G-I G-II G-III G-IV	10 7 2 17	11 14 19 4
Cobo F, et al. 1998 [9]	Liver	Non-registered information	G-I: ABZ (10 mg/kg/day 1 month before surgery) G-II: ABZ (20 mg/kg/day 1 months before surgery) G-III: ABZ (10 mg/kg/day) + PZQ (25 mg/kg/day 1 month before surgery)	Protoscolex viability. ABZ sulphoxide levels in serum and cyst fluid	Protoscoleces viability		
					G-III and G-I G-III and G-II		*p* = 0.004 *p* = 0.030
					ABZ sulphoxide levels		
					G-III and G-I G-III and G-II		*p* = 0.016 *p* = 0.034
Davis A, et al. 1986 [34]	Liver, lung, other organs	Non-registered information	MBZ (13 to 136.4 mg/kg/day) FBZ (37.5 to 54.5 mg/kg/day) ABZ (9.8 to 15.4 mg/kg/day)	Results: -success -partial success -improvement -no success	MBZ	FBZ	ABZ
					8 (9.4) 4 (4.7) 40 (47.1) 33 (38.8)	1 - - 5	5 (16.7) 4 (13.3) 14 (46.7) 7 (23.3)
Davis A, et al. 1989 [35]	Liver, lung, other organs	Non-registered information	ABZ (10 mg/kg/day 1 month) MBZ (1.5 to 4.5 g/kg/day, children half of the adult dose)	Results: -success -favourable effect -no success	Follow-up	ABZ	MBZ
					< 12 months: -success -favourable effect -no success no evaluation > 12 months: -success -favourable effect -no success	21 (100) - 13 (62) 5 (24) 3 (14) 46 (100) 18 (39) 18 (39) 10 (22)	23 (100) - 6 (26) 13 (57) 4 (17) 22 (100) 3 (14) 14 (64) 5 (23)
Franchi C, et al. 1999 [36]	Liver, abdomen, lung	Non-registered information	G-I: MBZ (50 mg/kg/day) G-II: ABZ (10–12 mg/kg/day) Both drugs in continuous 3- to 6-months cycles	Chest radiographic, ultrasonography, morphological changes and sonographic classification by *Caremani* et al	Cysts	G-I	G-II
					Treated Evaluated Changed Further deg. Relapse	289 271 152 34 37	640 611 502 110 134
Gil-Grande LA, et al. 1993 [37]	Liver or abdominal	G-A: 10.4 ± 4.1 G-B: 8.9 ± 4.3 G-C: 10.5 ± 5.1	G-A: ABZ (10 mg/kg/day 1 month before surgery) G-B: ABZ (10 mg/kg/day 3 months before surgery) G-C (control group): surgery (no ABZ treatment)	Protoscolex and cyst viability (patients/mice). Echographic changes	p-value		
					Viability of protoscolices 0.041 Intraperitoneal inoculation 0.167 Membrane disruption < 0.001 Echographic changes 0.038		
Keshmiri M, et al. 1999 [38]	Lung	E.gr: cm^3^ ± SD, 29.6 ± 50.5^All^ 27.1 ± 45.8^Treat.^ C.gr: cm^3^ ± SD, 18.3 ± 49.5^All^ 25.1 ± 63.3^Treat.^	Experimental group: ABZ (10–15 mg/kg/day 6 months) Control group: placebo	Radiological or ultrasonic findings. Response to treatment was classified: -Cured -Improved -No change -Worsened (*Caremani* et al*)*		ABZ	Placebo
					No. cysts Worse No change	124 9 (7) 32 (26)	26 10 (39) 13 (50)
					Decreased		
					> 25% (*p* < 0.001) > 50% (*p* < 0.001) > 75% (*p* = 0.067) Disappeared (*p* = 0.075)	83 (67) 60 (48) 36 (29) 16 (13)	3 (12) 1 (4) 1 (4) 0 (0)
Keshmiri M, et al. 2001 [39]	Lung, abdomen (including liver)	E.gr: cm^3^ ± SD, Lung, 29.6 ± 50.5^All^ 27.1 ± 45.8^Treat.^ Abdomen (liver), 198.1 ± 403.7^All^ 212.7 ± 426.2^Treat.^ C.gr: cm^3^ ± SD, Lung, 18.3 ± 49.5^All^ 25.1 ± 63.3^Treat.^ Abdomen (liver), 74.0 ± 130.8^All^ 91.9 ± 155.4^Treat.^	Experimental group: ABZ (400 mg twice a day, in 3 cycles of 6 weeks with 2 weeks between cycles) Control group: placebo	Volume. Ultrasonography and Computed tomography changes: 7 types, T1-T7. Response to treatment was classified: -Cured -Improved -No change -Worsened (*Caremani* et al)		ABZ	Placebo
					No. cysts Worse (*p* < 0.001) No change Improved (*p* < 0.001) Cure (*p* = 0.081)	172 15 (8.7) 23 (13.4) 117 (68) 17 (9.9)	31 11 (35.5) 16 (51.6) 4 (12.9) 0 (0.0)
Khuroo MS, et al. 1993 [40]	Liver	cm / cm^3^, mean ± SD At entry into the study vs the end of study PD, 9.2 ± 4.4 vs 5.1 ± 3.9 686 ± 651 vs 297 ± 515 ALB-PD, 10.8 ± 3.0 vs 4.8 ± 3.4 835 ± 528 vs 260 ± 389 ALB, 8.8 ± 4.5 vs 8.0 ± 5.0 642 ± 717 vs 606 ± 741	G-I: PD G-II: ABZ (10 mg.kg-1.day-1 for 8 weeks) plus PD G-III: ABZ alone	At entry into the study vs the end of study: -Clinical response -Cyst size -Cyst echopattern -Hydatid serology -Complications			*p*-value
					Clinical response		< 0.001
							< 0.005
					Cyst diameter Cyst volume Cyst echopattern Hydatid serology		< 0.05 < 0.01 < 0.01 NS
Mohamed AE, et al. 1998 [8]	1st, Liver(18), lung(1), multiple cyst(3). 2nd, Liver(13), lung(2), others:pelvis, mediastinum, kidney, spinal(4)	Non-registered information	1st, ABZ (400 mg twice day four weeks/two-week drug free period) 2nd, ABZ (400 mg twice a day) + PZQ (50 mg/kg)	Ultrasound and computed tomography changes, magnetic resonance, hydatid serology and chest-X-ray. Complete cure rates		ABZ	ABZ + PZQ
					No. patients	22	19
					Disappearance	8 (36.4)	9 (47.4)
					Liver		7/13
					Lung		2/2
					Reduction	5 (22.7)	9 (47.4)
					Liver		5/13
					Others		4/4
					No change	2 (9.1)	1 (5.2)
					Increase	0	0
Shams-UI-Bari, et al. 2011 [41]	Liver	Non-registered information	Group A: surgery. Group B: ABZ (10 mg/kg/day 12 weeks) + surgery + ABZ (10 mg/kg/day 12 weeks)	Viability, motility of the scolices and their ability to exclude 5% eosin, under immediate microscopy. Recurrence.		G-A	G-B
					Type I Type II Type III Type IV Viable Non-viable, *p* < 0.01 Recurrence, *p* < 0.05	12 (33.3) 10 (27.2) 8 (22.2) 6 (16.6) 36 (100) 0 (0) 6 (16.6)	11 (30.5) 11 (30.5) 10 (27.7) 4 (11.1) 2 (5.5) 34 (94.5) 0 (0)

^*^Statistical significance level of 5% (*p* < 0.05)

^a^*ABZ* Albendazole, *PZQ* Praziquantel, *MBZ* Mebendazol, *FBZ* Flubendazole

Most of the participants in the clinical trials were adult patients. The mean age (range age) of the participants was 36.7 (16–64) years in the Shams-UI-Bari et al. [41] study, and the maximum age was 52 (4–84) in the work of Franchi et al. [36]. The sample size of the studies varies, from 448 patients [36] to 15 [17] [others samples sizes were 121, 112, 84, 72, 55, 47, 41, 30 and 21 patients]. The number of cysts varied from 33 reported by Khuroo et al. [40] to 929 in Franchi et al. [35]. Most of the studies analysed multi-organ/abdominal cyts [8, 34–37, 39], liver [9, 33, 40, 41] and lung [38].

Several studies analysed albendazole alone [33, 37–41] or in combination with mebendazole [34–36] or praziquantel [8, 9]. Endpoints were protoscolex and cyst viability (viable/non-viable or intact/dead), and/or response to treatment (cured, improved, no changed, worsened). Due to differences among articles, we could not extract data for statistical treatment from 4 out of 11 papers. In the study by Franchi et al. [35], cysts treated with albendazole relapsed in 134/640 (20.9%) cases, while the rate for mebendazole treatment was 37/289 (12.8%). In the case series by Keshmiri et al. 1999 [38] and 2001 [39], only one case relapsed following albendazole treatment (1/11; 9.1%), and 1/17 (5.8%) relapsed following mebendazole treatment. According to Shams-UI-Bari et al. [41], patients who did not receive albendazole therapy reported a recurrence rate of 6/36 (16.6%), while no recurrence was reported in patients who received albendazole therapy (*p* < 0.01). Albendazole therapy was associated with fewer recurrences than mebendazole therapy.

Seven studies were examined in three meta-analyses, as described below.

**Fig. 2:**
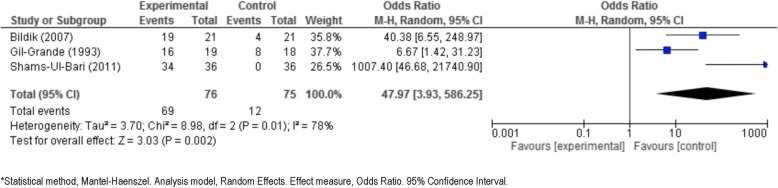
Forest plot of comparison: intervention (albendazole plus surgery) vs control (surgery alone), outcome: Viability of scolex (Event = non-viable or dead)

**Fig. 3:**
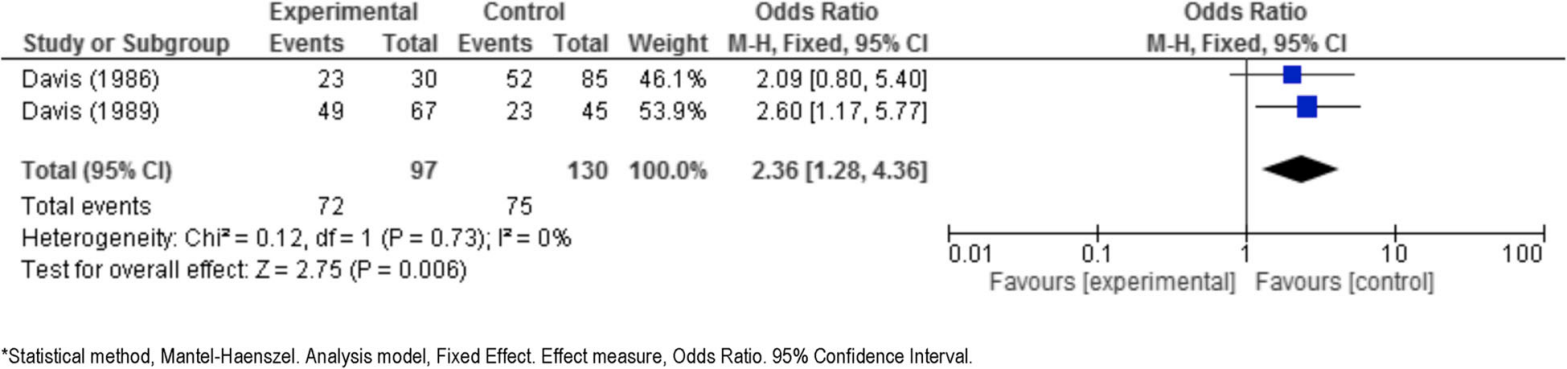
Forest plot of comparison: intervention (albendazole) vs control (mebendazole), outcome: Response to treatment (Event = cure/success plus improvement)

**Fig. 4:**
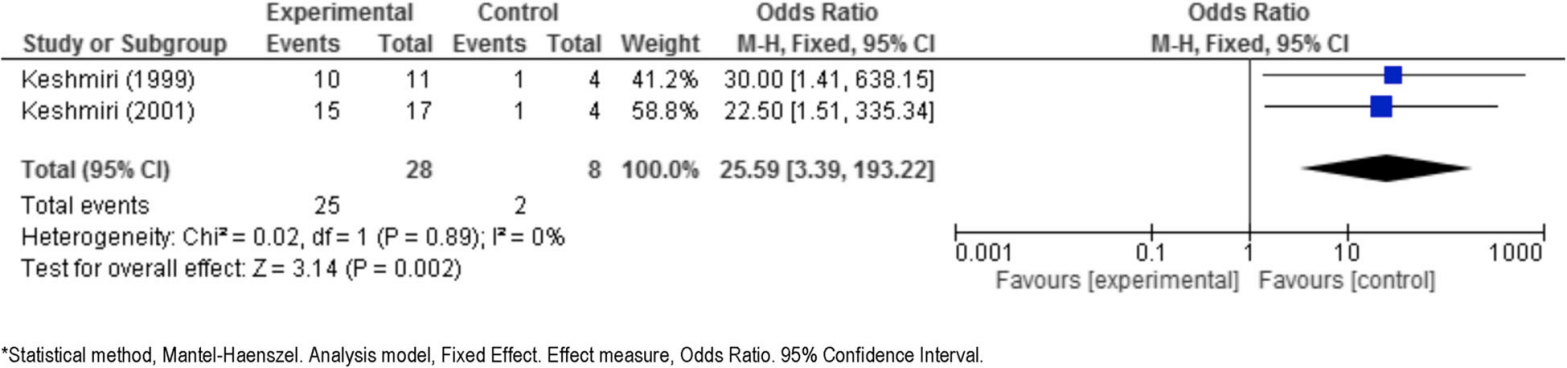
Forest plot of comparison: intervention (albendazole) vs control (placebo), outcome: Response to treatment (Event = cure/success plus improvement)

i) *Albendazole plus surgery* versus *surgery alone.* Blidik et al. [33], Gil-Grande et al. [37], Khuroo et al. [40], and Shams-UI-Bari et al. [41] address this issue. These studies compare albendazole plus surgery to surgery alone and analyse the viability of the scolex as a common point. In all 3 studies, the number of non-viable scolex in the experimental group (albendazole plus surgery) is higher than the control group (surgery alone) (Tables 3 and 4). These three studies were significantly heterogeneous (*p* = 0.01 for Q test and 78% for I^2^), so a random-effects model was used. The results are shown as a forest plot in Fig. 2.
  The summary odds ratio was 48, which is clearly significant in favor of albendazole plus surgery, with a *p*-value of 0.002 according to the Z test.
  Davis et al. 1986 [34], Davis et al. 1989 [35], Keshmiri et al. 1999 [38] and Keshmiri et al. 2001 [39] answer the second clinical question that we considered in this meta-analysis*:*
ii) *Albendazole* versus *mebendazole*. These studies compare albendazole to mebendazole (Fig. 3) and analyse the response to treatment (cured, improved, no changed, worsened) as a common point. In all studies, the proportion of cysts cured/success or improvement in the experimental group (albendazole) is higher than that in the control group (mebendazole or placebo) (Table 4).
  Figure 3 (forest plot) shows the results [34, 35]. The Q test of heterogeneity was not significant (*p* = 0.73), indicating excellent homogeneity (I^2^ = 0%) of these studies.
iii) *Albendazole* versus *placebo.* These studies compare albendazole to placebo and analyze the response to treatment (cured, improved, no changed, worsened) as a common point. In both studies, the proportion of radiological cured/success or improvement in the experimental group (albendazole) is higher than that in the control group (placebo). Figure 4 shows the results obtained from comparing albendazole to placebo [38, 39].
  The test of heterogeneity was not significant (*p* = 0.89), indicating excellent homogeneity (I^2^ = 0%).
  Cobo et al. [9], answer the third clinical question that we considered in this meta-analysis*.*
iv) *Albendazole* versus *albendazole plus praziquantel*. It was not possible to statistically combine the data comparing albendazole plus praziquantel treatment to albendazole alone. However, an answer is given in Mohamed et al. [8] and Cobo et al. [9]. In the latter paper [9], the number of non-viable scoleces in the albendazole plus praziquantel group was higher than in the albendazole alone group. According to Mohamed et al. [8], the reduction in the number of cysts and cases cured or improved in the albendazole plus praziquantel group was higher than it was in albendazole alone group (Table 4). It was not possible to generate a forest plot.

## Discussion

The clinical handling of CE involves four therapeutic alternatives: surgery, percutaneous intervention, drugs, and the “watch and wait” approach for quiescent cysts. The evidence supporting pharmacological treatment is weak. This lack in quality is due to i) small number of patients forming an homogeneous clinical group, even in referral hospitals in endemic countries; ii) the different applied methodology prevents comparison between studies; iii) CE is a chronic disease, which requires long-term monitoring to determinate the effectiveness of an intervention [42]. There are no gold standard methods to determinate biological status and response to treatment [37]. Drug-induced echographic changes can be compare with viability studies of protoscoleces developed on surgically removed cysts, which would generate parasitological data to correlate with clinical effects. These objectives were used by Gil Grande et al. [37] in their randomized controlled trial of the efficacy of albendazole, but data collection has not been posible for praziquantel, despite its clinical use in last 20 years. Furthermore, hydatid cysts may spontaneously regress with shedding of membranes, solidifying and calcification in their natural history, without any chemotherapeutic assistance [43].

Despite attempts by the WHO, the management of CE disease remains a major problem [44]. There is no consensus on disease management [1, 45]. Thereby, studies to establish the efficacy of medical treatment, the dosing and the minimal effective dose have not been yet determined by sufficient evidence. Case definitions, diagnostic proceedings and defined monitoring methods for long-term follow-up need to be standardised, and comparative efficacy surveys need to be performed. Future progress in chemotherapy may be attained by identifying drugs with higher anti-echinococcal activity.

### Clinical question 1

#### Surgery/PAIR vs surgery/PAIR plus albendazole/mebendazole

Our data show that treatment outcomes are better when surgery or PAIR is combined with chemotherapy of benzimidazole drugs given pre- and/or post-surgery. The summary odds ratio found in the meta-analysis shows a value of 48 (95% CI: 4–586), which is far greater than the value of 1 that would indicate equality of the treatments.

Chemotherapy is applied in many scenarios, so it is very difficult to standardize the results. Anthelmintics are usually indicated before and after surgery to reduce the size of cysts, to sterilise them, and to prevent relapses. Furthermore, medical treatment is the only therapeutic option in scattered CE and/or inoperable CE. To date, there are insufficient data to establish the optimal duration of treatment or frequency of dose. Recommendations on the timing of the start of chemotherapy before surgery or PAIR are varied. Preoperative treatment with albendazole begins at least 3 months to 1 day before surgery and/or PAIR and continues for 1–3 months post-treatment, and there is no clear recommendation on praziquantel dosage schedules. In terms of cyst viability and radiological efficacy, the data are not conclusive as to whether longer courses of treatment (3 months) are more efficacious than shorter courses of treatment [19, 37].

The additional benefit from very long treatment (more than 6 months) is marginal for most patients, and although it is performed in clinical practice with patients with multiple or inoperable CE, it has never been well evaluated. There is a suggestion that lesions in organs other than the liver, lung and peritoneum may benefit from more prolonged therapy, but the numbers are small.

### Clinical question 2

#### A*lbendazole* vs. *mebendazole*

Our data show in all of the studies that the odds ratio of cure/success or improvement in the albendazole group is greater than those in the other groups (mebendazole or placebo). To be exact, the summary odds ratio for albendazole versus mebendazole was 2.4 (95% CI: 1.3, 4.4) with a *p*-value of 0.006. There is some evidence that albendazole should be chosen over mebendazole, at least until more trials are reported. As expected, the comparison of albendazole and placebo is in favor of the drug treatment, with a p-value of 0.002 for the Z test of significance.

Mebendazole is a broad-spectrum antihelminthic agent of the benzimidazole type with in vivo activity in CE. Nevertheless, albendazole is more active in vitro than mebendazole and has better gastrointestinal uptake and bioavailability [46]. It has been published better clinical outcomes with albendazole [47]. Flubendazole is not already used for hydatidosis. Testing hepatic enzymes and blood count every 2 weeks is recommended during benzimidazole treatment to monitor most frequent adverse effects [48]. Although it is orally administered, albendazole results in high serum concentration but penetration into cyst is patchy. Albendazole and mebendazole may reduce volume of hydatid cysts and may become them sterile in some cases [47]. However, less than half treated patients achieve clinical and radiological resolution without concomitant drainage [47]. Albendazole, with or without PAIR, is usually given in two divided doses (10–15 mg/kg/day) or 400 mg once a day. Daily dose of mebendazol is 40–50 mg/kg in three divided doses. Treatment may be administered in several cycles (1–6) separated by 10–14 days. Clinical and radiographic improvement is frequently noticeable. Radiological endpoints are commonly defined as more than 25% decreases in cyst size, membrane detachment or cyst calcification. Cyst disappearance occurs in less than 50% of patients in medical treatment [49]. Our data show that the efficacy of albendazole was superior to that of mebendazole, with a *p*-value of 0.009. Although there are works that show that the rate of relapse was similar for patients treated with albendazole or mebendazole, it is necessary to carry out prospective studies over long periods to generate robust data.

### Clinical question 3

#### Albendazole vs albendazole plus praziquantel

Our data show that combined treatment with albendazole plus praziquantel is superior to treatment with albendazole alone.

Praziquantel, an isoquinolone derivative, has had limited use in the treatment of CE [47]. Praziquantel has shown efficacy in vitro and in animal models. Weekly dose of 50 mg/kg or dose every 2 weeks have displayed suitable pharmacokinetics in humans [47]. There are few clinical studies documenting praziquantel benefits in humans [8, 9, 30, 50]; however, combination of praziquantel with mebendazole [50] or albendazole [8, 9] seems to be more effective and possibly more rapid than benzimidazole monotherapy. The earliest report of a trial of combination praziquantel and albendazole in the treatment of human hydatid disease was made by Yasawy et al. [30]. Major disadvantages of these works are different praziquantel schemes: various treatment groups are too small for significant analysis and failure to use matching controls [8, 30]. Combination of praziquantel and albendazole may produce some benefit in pre- and post-intervention chemotherapy and might be helpful when spillage occurs during surgical procedure [8, 9]. Combined treatment reduces potentially the risk of disease recurrence and intraperitoneal seeding of infection that may develop via cyst rupture and spillage. Additionally, praziquantel may prevent the vesicular development of protoscoleces and inhibit the formation of secondary cysts. Combination therapy increases levels of albendazole sulphoxide (the active metabolite of albendazole) both in serum and in cyst fluid compared with levels in patients received only albendazole [14]. Praziquantel has been given at a dose of 40 mg/kg in different regimens for each patient (daily, weekly, fortnightly or monthly) with standard courses of albendazole for between 2 and 3 months. At present, there is scarce evidence supporting a recommendation for the routine use of praziquantel in prolonged chemotherapy for established CE where surgery is not indicated or in severe disseminated disease. This treatment and dosage regimen require evaluation. Further randomized controlled studies are required to determine whether there are significant advantages of combination therapy with albendazole and praziquantel to clarify treatment recommendations. Finally, there are few medical therapeutic options available for CE. As there is some evidence of usefulness of praziquantel in this disease, potential benefits should be explore.

### Limitations

One of the limitations of this study was the calculation of *overall effects* in the meta-analysis sections due to the scarcity of data. Some authors argue that, since clinical and methodological diversity always occurs in meta-analyses, a good statistical combination of studies is always difficult [51, 52]. Nevertheless, a qualitative review and a meta-analysis are better than a lack of information. The authors used *forest plots* to interpret the results of the meta-analyses, which is an accepted methodology. However, when there are few studies, these plots and their associated tests of significance are not very robust, and more studies are necessary to obtain conclusive evidence.

Risks of bias (methodological and clinical) may have a bearing on the results of our qualitative review and meta-analysis. The overall effect of meta-analysis may be affected by publication bias, overestimating the efficacy of treatment, since studies with statistically significant results are more likely to be published than those with non-significant differences. Funnel plots visually check the possible existence of publication bias, but unfortunately, this type of analysis cannot be used when the number of included studies is scarce, as it was the case here.

Despite these limitations, this systematic review and meta-analysis seeks to synthesize the large volume of information available up to date related to medical treatment of CE to help make decisions on evidence-based medicine in daily clinical practice.

## Conclusions

### Does our study provide sufficient evidence to influence decisions for the treatment of CE?

In the opinion of the authors, this analysis of the literature suggests the following claims: i) pharmacological treatment improves results in patients with CE who will undergo surgical treatment; ii) for now, albendazole chemotherapy is the primary medical treatment to consider in the medical management of CE; and iii) treatment with albendazole plus praziquantel shows higher scolicidal activity and a greater number of cysts cured or improved compared to albendazole alone.

## Additional file

Additional file 1: Search strategy in database. (DOCX 15 kb)

## Abbreviations

CE: Cystic echinococcosis
CONSORT: Consolidated standards of reporting trials
OCEBM: Oxford centre for evidence-based medicine
PAIR: Puncture, Aspiration, Injection of protoscolicidal agent and Reaspiration
PRISMA: Preferred reporting items for systematic reviews and meta-analyses
WHO: World Health Organization
