# Supplementary material for: Medical treatment of cystic echinococcosis: systematic review and meta-analysis
Source: BMC Infect Dis. 2018 Jul 5;18:306. doi: 10.1186/s12879-018-3201-y (PMC6034244; doi:10.1186/s12879-018-3201-y)
Supplement: Supplementary file 1 — Search strategy in database. (DOCX 15 kb) [file 12879_2018_3201_MOESM1_ESM.docx]

**SUPPLEMENTARY METHODS**

The search strategy was as follows:

1. "hydatid disease"[All Fields] OR "cystic echinococosis"[All Fields]
2. "*Echinococcus granulosus*"[All Fields]
3. #1 AND #2
4. "medical treatment"[All Fields]
5. "albendazole"[All Fields] OR "mebendazole"[All Fields] OR "praziquantel"[All Fields]
6. #4 OR #5
7. #3 AND #6
8. #7 AND "randomized controlled trials"[All Fields]
9. #8 AND "humans"[MeSH Terms]

The filter *Etiology/Broad* was applied through the *Clinical Queries* tool.
